# Trends in National-Level Governance and Implementation of the Philippines’ Responsible Parenthood and Reproductive Health Law from 2014 to 2020

**DOI:** 10.9745/GHSP-D-21-00184

**Published:** 2021-09-30

**Authors:** Vanessa T. Siy Van, Jhanna Uy, Joy Bagas, Valerie Gilbert T. Ulep

**Affiliations:** a Health Sciences Program, Ateneo de Manila University, Quezon City, Philippines.; b Philippine Institute for Development Studies, Quezon City, Philippines.

## Abstract

In 2012, the Philippines passed the Responsible Parenthood and Family Planning Law, a landmark legislation billed as a multisectoral and rights-based approach to further sustainable human development. This article is part of the first comprehensive evaluation of the implementation of the law by national-level actors. This evaluation is intended to assess the progress of implementers in the conduct of mandates, roles, and responsibilities described in the law and its implementing guidelines. Interviews with key national government officials and data from official documents and literature revealed 3 major trends in governance and implementation from 2014 to 2020. First, despite being a multisectoral policy, performance was siloed within individual units of implementing agencies, with limited interagency coordination. Second, although the law explicitly called for interventions to invest in human capital and address socioeconomic disparities for sustainable human development, performance focused on biomedical and health interventions, particularly in the area of family planning. Third, national-level governance for reproductive health interventions concentrated on programmatic and operational concerns. Overall, this case in the Philippines illustrates that fragmented implementation has contributed to the slow improvement of reproductive health outcomes. This study highlights the challenges of governance and multisectoral coordination to implement multidimensional interventions in a low- and middle-income country, and it provides potential areas for political and administrative reform in reproductive health governance in the Philippines. By creating a common narrative and onboarding multiple sectors, officials can better identify and address structural determinants with holistic policy solutions to improve reproductive health outcomes.

## Abstract (precis)

National-level implementation of the Philippines’ Responsible Parenthood and Reproductive Health Law has been fragmented and programmatic and centered on family planning rather than multisectoral and holistic. Establishing a common narrative can secure the buy-in of different sectors and open policy solutions to address the structural determinants of reproductive health.

## Teaser

**Key Messages:**

- The Philippines’ Responsible Parenthood and Reproductive Health Law acknowledged reproductive health as a multisectoral initiative. However, national-level implementers of the law had little intersectoral collaboration and focused on specific biomedical programs that were insufficient for remarkably improving reproductive health outcomes.

**Key Implications:**

- Policy makers must shift away from a health-centric agenda anchored on family planning policy and aim toward holistic life-course interventions.
- National health leaders must secure other sectors’ interest in reproductive health rather than rely on weak health-centric leadership based on law alone.
- Government agencies should establish a common narrative to engage different sectors to view reproductive health holistically and contribute multisectoral policy solutions.

## INTRODUCTION

### Multisectoral Governance for Reproductive Health

Beyond biological health, reproductive health (RH) is tied to access to information and services that enable choices for the well-being of one’s self and family.^1^ From empowering individual and household decisions, comprehensive RH policies have national impact, facilitating sustainable population growth, human capital investment, and socioeconomic development.^2^^–^^4^

To effectively accomplish these goals, the provision of acceptable and affordable RH care services requires an awareness of the social and economic determinants^5^^–^^7^ affecting gender and interpersonal relations. Hence, interventions to address the social determinants of sexual health and RH must acknowledge that individual health outcomes are not solely determined by the health sector but instead require action from multiple sectors.^8^

A critical factor for the success of multisectoral action is governance,^9^ which encompasses how activities of different stakeholders with respective responsibilities and resources can be oriented toward a single vision in a process of negotiation, collaboration, and reporting for enforcing accountability.

Governance plays an important role in low- and middle-income countries (LMICs),^10^ creating a policy environment conducive to investing in social development without stifling economic growth. Amid the difficulties of this dual role, plans are affected by limited resources and competing political interests.^11^ Thus, many LMICs have yet to implement successful multisectoral governance approaches.^9^

### Structure of RPRH Implementation

The Philippines passed the Responsible Parenthood and Reproductive Health (RPRH) Law in 2012, after years of opposition from conservative groups.^12^^,^^13^ It declared universal access to RH services as integral to the rights to life, health, and sustainable human development.^14^

The wide scope of RPRH envisioned contributions from multiple sectors represented by national government agencies (NGAs) (Supplement). In late 2014, the National Implementation Team (NIT) was created to manage and coordinate interagency RPRH activities.

NIT is chaired by an undersecretary of the Department of Health (DOH), and its members include NGAs, civil society organizations (CSOs), and multilaterals. Keeping with the decentralized system of governance in the Philippines,^15^ regional implementation teams, composed of government agencies’ regional office staff, were envisioned to mirror NIT’s functions at the regional and local government unit (LGU) levels.

## RATIONALE AND OBJECTIVES

This study was part of the first evaluation of the RPRH Law, commissioned by the Philippine DOH. The study objectives aimed to:

- Assess the progress of NGAs across sectors in the conduct of mandates, roles, and responsibilities described in the RPRH Law and its Implementing Rules and Regulations (IRR).^16^ Analyses focused on the trends in national-level governance and implementation of the law.
- Provide information on multisectoral governance in an LMIC context, where most multisectoral action for health pertains to communicable diseases, maternal health, and nutrition.^17^^,^^18^
- Assess national implementers’ performance over 7 years, from the perspective of different sectors, which is especially relevant for overcoming limitations of policy and implementation lags and accounting for externalities that health-sector governance may have on other sectors.
- Present pragmatic recommendations for engaging multiple sectors in health policies.

The study assessed the progress of individual NGAs and across sectors regarding conduct of mandates, roles, and responsibilities described in the RPRH Law and its IRR.

## METHODS

### Defining Governance

An operational definition of governance was necessary to situate national-level implementers’ conduct of their respective mandates and inform the data collection process. Governance^19^ refers to the formal structures and processes that promote inclusive participation, responsiveness to people’s needs, and accountability among stakeholders. Governance also includes informal processes and power dynamics that facilitate multistakeholder coordination to achieve public goals. Taken together, these formal and informal decisions^20^ are the policies that enable the timely and appropriate provision of social services.

Governance refers to the formal structures and processes that promote inclusive participation, responsiveness to people’s needs, and accountability among stakeholders.

### Data Collection and Analysis

Data collection focused on the RPRH activities of agencies involved in NIT. As NIT does not have its own staff, member agencies send representatives for monthly meetings.

Twenty key-informant interviews (KIIs) were conducted with respondents involved in RPRH governance at the national level (Table 1) who held positions within their organizations of program managers or higher (Supplement).

**TABLE 1.** Organizations Included in Key Informant Interviews and Number of Respondents

**Organization**	**Respondents**
National Implementation Team	1
Department of Health	6
Commission on Population and Development	4
Department of Social Welfare and Development	2
Philippine Commission on Women	2
Philippine Health Insurance Company	1
Department of Education	1
Department of Interior and Local Government	1
Civil society/private sector	1
United Nations Population Fund	1

Most interviewees were career bureaucrats who had worked in government for a decade before promotion to their current position. At the time of the study, they had been in their current positions between 1 and 15 years, with a median of 4 years of experience.

KIIs with other implementing agencies were planned, but representatives did not respond to the interview invitation letters. Follow-ups for these agencies were not pursued further due to the onset of the COVID-19 pandemic and the acquired information reaching data saturation.^22^ No respondents dropped out of the study.

### Key Informant Interviews

The KIIs explored how agencies adapted their organizational structures and roles for RPRH activities based on their mandates. NGAs were asked to identify and self-evaluate their performance on RPRH-related activities. Implementing agencies were also asked about their role in NIT and their organizations’ participation in NIT meetings. They then enumerated major challenges to accomplishing their mandates and implementing the RPRH Law in general. Interviews were audio-recorded with the consent of participants and transcribed verbatim. In the findings section, we present translated quotes from KIIs and redact portions of the translations that may lead to identification of the respondents. From the transcripts, patterns, trends, similarities, and differences in answers were identified and analyzed independently by 3 researchers using qualitative thematic analysis and synthesized to form an assessment of overall performance.

A review of official documents confirmed whether agencies’ mandates were implemented. These included the RPRH Law, IRR (2017 revision), policy issuances after 2013, joint circulars, and executive and administrative orders. Also scoped were the annual accomplishment reports (ARs) published by the NIT secretariat, based on RPRH implementation reports submitted by member agencies. The reports summarize performance, challenges, and recommendations for 5 chosen key result areas (KRAs) as well as governance and financing. Seventy-one of 79 NIT meeting transcripts from the years 2014 to 2019 were also obtained for analysis.

These data were triangulated with a review of secondary literature. The review included studies that documented or evaluated the activities of implementing NGAs for RPRH. Additional literature validated whether findings from KIIs and official documents were common in the setting of Philippine governance or multisectoral governance. Finally, the literature review also determined best practices and recommendations that may contribute to solving challenges in RPRH governance based on cases from similar contexts.

## FINDINGS

This section presents 3 interconnected findings.

1. Implementation activities were siloed to individual agencies and compartmentalized to specific programs and policies.
2. Siloed programs and processes, coupled with the historical and political context of the law pushed health-sector activities to the forefront of implementation, leading to a focus on biomedical, particularly family planning (FP), interventions.
3. NIT meetings concentrated on program-specific issues brought up by prominent health-sector agencies, which steered implementation toward being programmatic and operational.

The study revealed that implementation activities were siloed, interventions were heavily focused on FP, and implementation tended to be programmatic and operational.

### Siloed and Compartmentalized Performance

Agencies were generally able to fulfill their mandates; however, these mandates did not require interagency coordination or coordination between different divisions of the same agency. Table 2 qualitatively evaluates the completion of RPRH mandates assigned to NGAs as “not done” (to be implemented), “doing” (some implementation activities), or “done” (completed). Siloed performance was observed both among and within agencies. A significant portion of completed mandates were those that were one-time, straightforward tasks. The majority of these were assigned to DOH, such as the creation of guidelines and standards.

**TABLE 2.** Completion of RPRH Mandates Listed in RPRH Implementing Rules and Regulations (2017 Revision) by Subsection^a^

**Done**	**Doing**	**Not Done**
**DOH Governance: Guidelines**		
**4.01** Service Delivery **4.04** Informed Choice **4.08** Care for GBV Survivors **4.12, 4.13** Policies on Life-Saving Drugs in Maternal Emergencies **5.07** FP Services at Establishments/Enterprises **5.08** Mapping Service Delivery Network (SDN) Facilities **5.22, 5.23** Exempting Private Providers **6.01, 6.03-6.05** Contracting and Training Health Professionals **8.03** Procurement of FP **10.10** LGU Awards/ Recognition **14.01** Maternal, Fetal, and Infant Death Reviews (M/FIDR)	**5.07** FP Services at Establishments/Enterprises **5.11** Match Populations to Facilities in the SDN **5.14** Assistance to LGUs for Mobile Health Care Services (MHCS) Vehicles **5.17** Identification of Facilities for Upgrading **5.19** Support to LGUs **5.27** Training Counselors of Adolescents **6.06** Comprehensive Emergency Obstetric and Newborn Care (CEmONC) Curriculum **6.07, 6.08** Training Barangay (Community) Health Workers **10.02** Health Promotion Plan **12.01 except section h** DOH duties	**5.13** Standards of MHCS **5.18, 5.20** M&E for LGU Fund Utilization and SDN **6.02** Determine number of Skilled Health Professionals **10.06** M&E for New Health Promotion Plans
**DOH Governance: Non-Guidelines**		
**5.09, 5.10** Mapping Health Facilities and Priority Populations in the SDN **8.02** Budget to Procure FP **9.03, 9.04** Funds for Health Facilities and Public Awareness	**6.10** Technical Assistance to Engage Private Providers in LGUs **14.07** M/FIDR Panel **15.03** Streamline Reporting	**8.07, 8.10** Monitoring System for Procurement **12.01-h** RH Bureau in DOH
**DOH Service Delivery**		
**4.11** Life-Saving Drugs in Maternal Care Emergencies **4.15** Maternal and Newborn Health Care in Crisis Situations **5.21** Assist Private FP Services **7.02, 8.01, 8.08** FP Logistics **13.02** RPRH in Anti-Poverty **15.01** Reporting Requirements	**4.05-4.07** Access to FP (including minors) **4.09** PWD-RH Programs **4.10** Responding to Unmet Need **5.02, 5.05** RH Care in SDN **5.12** MHCS **6.09** SBCC Materials **10.01** Health Promotion	
**Other Implementers**		
**5.10** Identify Priority Populations in the SDN (**DSWD**) **12.03, 12.04** Duties (**DSWD**) **7.04, 7.05, 7.08, 7.12** RH Product Certification (**FDA**) **7.06** Harmonize Standards (**FDA**) **9.06-9.08** Financing RH (**PhilHealth**) **10.07, 12.04** CSO Participation (**Cross-Cutting**) **11.02** Curriculum Development (**DepEd**)	**4.02**, **5.03, 5.15, 5.16, 8.09, 10.05** Service Delivery (**LGUs**) **5.07** FP Services at Establishments/Enterprises (**Department of Labor and Employment**) **9.01, 9.02** Appropriations (**Cross-Cutting**) **10.04** NGAs Assist DOH **10.08, 10.09** Health Promo in NGAs’ Programs (**Cross-Cutting**) **9.05** Funding for RPRH Education (**PRC/CHED/TESDA/DepEd**) **11.01, 11.05, 11.06** Provision of RPRH Education (**DepEd**)	**4.14** Integrate RH in Health Professional Curriculum (**PRC/CHED/TESDA**) **6.11-6.13** Pro-Bono Services Requirements (**PhilHealth**) **7.07** Guidelines for FP Product Requirements (**FDA**) **7.09, 7.10** Post-Marketing Surveillance Unit (**FDA**) **11.04** Training Educators (**DepEd**)

Abbreviations: CHED, Commission on Higher Education; CSO, civil society organization; DepEd, Department of Education; DOH, Department of Health; DSDW, Department of Social Welfare and Development; FDA, Food and Drug Administration; FP, family planning; GBV, gender-based violence; LGU, local government unit; M&E, monitoring and evaluation; NGA, national government agency; PhilHealth, Philippine Health Insurance Corporation; PRC, Professional Regulation Commission; PWD, persons with disabilities; RH, reproductive health; RPRH, Responsible Parenthood and Reproductive Health; SBCC, social and behavior change communication; TESDA, Technical Education and Skills Development Authority.

^a^Sources: RPRH Implementing Rules and Regulations (2017 revision), annual accomplishment reports, key-informant interviews, and review of official documents and secondary literature.

For some agencies, completed mandates aligned with their existing agency functions and could be accomplished without other implementers. An example is the Food and Drug Administration’s (FDA’s) role in certifying FP commodities for the Philippine National Drug Formulary and Essential Medicines List. Similarly, Philippine Health Insurance Corporation (PhilHealth), with its core function of benefit package development,^23^ fulfilled its mandate of having packages and case rates covering HIV/AIDS, breast and reproductive tract cancers, menopause-related conditions, and long-acting and permanent contraception.

In contrast, although few mandates explicitly mention overlap between the roles of 2 or more agencies, these made up most of the partial or incomplete accomplishments. An example is the compartmentalized co-management of the national FP program (NFFP) supply chain. In the backend, DOH is responsible for procuring FP commodities but has not yet established a computerized procurement tracking system. This contributes to delays in the FDA’s overseeing the adherence of FP suppliers to proper handling, storage, and distribution and the conduct of post-marketing surveillance. The Commission on Population and Development’s (POPCOM’s) and LGUs’ roles in FP distribution are the front-end components of the program’s service delivery network. However, weak coordination with DOH has led to recurring supply-demand mismatches between procurement and distribution. In 2018, the Commission on Audit^24^ found excessive procurement and overstocking of FP commodities, with many undelivered and expired. Their investigation found that DOH’s poor inventory management was caused by weak and inefficient coordination within DOH central office units and between DOH central office and regional offices.

Within agencies, initiatives whose entire project cycle was handled by a sole implementing unit were mostly completed. For instance, the conduct of maternal, infant, and fetal death reviews fell under the oversight, evaluation, and support functions of the DOH Safe Motherhood Program.

However, compartmentalization within individual agencies has also contributed to incomplete accomplishments. DOH has still not been able to formulate annual monitoring and evaluation (M&E) plans, targets, and resources for its national multimedia campaigns, given that 3 separate bureaus are responsible for various inputs such as the technical content, material design, and M&E indicators. Another example is the rollout of the Department of Education’s (DepEd’s) comprehensive sexuality education curriculum, which the DepEd respondent rated only 40% implemented after 5 years of work. Because each change to the curriculum requires approval from 4 bureaus, implementation experienced significant delays. Both DOH and DepEd respondents said bureaucratic delays were an inefficiency that hindered agency performance.

### Biomedical and FP Focus

Despite the multidimensional nature of RPRH, FP received disproportionately more efforts and resources from RPRH implementers than did any other element. FP has been brought up in almost every single NIT meeting, while the next most frequent elements are raised only half the time (Table 3). Moreover, FP programs are nationwide in scope with sustained implementation. As a result, other RPRH elements received less attention. For instance, the Mental Health Act of 2018 did not prompt discussions around Element 12 leading up to and following its passage.

**TABLE 3.** NIT Agenda by RPRH Element and Frequency in 71 NIT Meetings From 2014 to 2018^a^

**RPRH Element**		**Frequency**
1	Family planning	60
2	Maternal, neonatal, child health, and nutrition	32
11	Adolescent reproductive health (RH) education in formal and nonformal settings (i.e., comprehensive sexuality education)	30
4	Adolescent youth and RH guidance and counseling	27
3	Proscription of abortion and management of abortion complication	15
7	Sexuality and RH education and counseling	14
6	Violence against women and children and gender-based violence	12
5	Sexually transmitted infections, reproductive tract infections, and HIV/AIDS	11
8	Breast and reproductive tract cancers and other gynecological conditions and disorders	3
9	Male responsibility and involvement and men’s RH	2
10	Prevention, treatment, and management of infertility and sexual dysfunction	1
12	Mental health aspect of reproductive care	0

Abbreviations: NIT, National Implementation Team; RPRH, Responsible Parenthood and Reproductive Health.

^a^Source: NIT Meeting Transcripts 2014–2018.

Despite the multidimensional nature of RPRH, FP received disproportionately more efforts and resources from RPRH implementers than did any other element.

The preponderance of FP may be partially attributed to the 5 KRAs chosen by NIT in 2015. These focused implementations on maternal, neonatal, and child health and nutrition, FP, adolescent sexual and reproductive health, violence against women and children, and sexually transmitted infections and HIV/AIDS. As such, most programs and initiatives reported in the annual ARs fell under RPRH elements corresponding to the 5 KRAs chosen by NIT (Supplement).

Although FP did not have the greatest number of unique programs, all 4 programs are regularly implemented in hundreds of sites throughout the country. Family counseling sessions are a requirement for those seeking marriage and applying for social welfare programs.^25^ Businesses are also required to provide FP services to operate. In contrast, adolescent sexual and reproductive health has the most reported programs (Elements 4, 7, 11), but the majority of these were one-off events such as forums, competitions, seminars, and modules. FP programs are also explicitly required by the RPRH Law, whereas programs in other KRAs predate the RPRH Law and were created in response to pre-existing laws.

Even before the KRAs, FP came to the forefront of RPRH implementation and multi-agency efforts as implementers felt this area faced the most challenges. While other RPRH programs had longstanding support from elected officials, FP had a long history of controversy. Years of struggling to pass the law were due to the provisions related to FP. Just 4 days after the signing of its IRR in 2013, a Supreme Court status quo ante order delayed its implementation over a year, calling into question the constitutionality of FP services.^26^ From 2015 to 2017, the Supreme Court imposed a temporary restraining order on the certification of contraceptive products by the Food and Drug Administration (FDA) and the procurement, distribution, and use of the progestin subdermal implants.^27^ The current FP-centric approach taken by implementers was bolstered by support from the Office of the President in 2017^28^ and renewed in 2019^29^ to fully implement NFFP as part of the country’s medium- and long-term development plans,^30^ cementing FP’s place as the linchpin of RPRH implementation.

*We don’t feel the weight of other programs because the NIT meetings rarely touch on them. They are so focused on family planning. I attended 1 forum. Another attendee asked me why they always talk about family planning. So, I just answered, FP is a big problem because a large sector is against it.* —Respondent 10

National implementers’ focus on FP from the days of lobbying for the law, overcoming legal obstacles, and capitalizing on current executive support have made the DOH, Commission on Population and Development (POPCOM), DepEd, PhilHealth, and CSOs the most visible actors for RPRH. Interviews with NGA representatives found that even among NGAs, RPRH is considered DOH’s responsibility, and DOH is viewed as the leader of implementation.

However, deferring to the health sector emphasized a biomedical view for RPRH implementation. One anecdote from the KIIs highlighted the focus on and insufficiency of biomedical interventions to reduce adolescent pregnancy and maternal mortality. Instead, social determinants directly influenced young mothers’ health outcomes. Although some adolescent mothers were children of doctors, they delayed seeking medical care and informing their parents. A failure to address underlying determinants such as parental education, sensitivity training for health workers, and male responsibility in RH diminished the effectiveness of fertility management programs. This reality stands in contrast to the paradigm that population management alone can address all RH issues, which is still espoused in national policy.

*So mainly it’s about family planning… that is because of the mandate of the president. The president is connecting family planning with poverty alleviation. So, it’s not just a health concern, but about alleviating poverty. On teenage pregnancy, to be declared by the president as a national social emergency, it is still related to family planning. The president is also saying that in his State of the Nation Addresses.* —Respondent 11

The prominence of biomedical discussions has failed to secure the buy-in of NGAs outside the health sector to play a bigger role in RPRH implementation. This is exemplified by the absence of their agencies in NIT meetings and low attendance from higher-level decision makers.

### Programmatic and Operational Concerns

National RPRH governance has focused on service delivery, specific programs, and micro-operational concerns above multisectoral governance and leadership. Persistent challenges and recommendations identified in the annual ARs (Table 4) and KIIs highlighted the absence of legal, financial, and M&E mechanisms to support agencies’ initiatives across all KRAs. For specific KRAs, governance challenges in planning, priority setting, coordinating, and combating stigma were brought up only in later years. Recommendations given to address these issues remained siloed within individual programs and agencies. Moreover, the KRA-based annual ARs lacked systematic evaluation of progress against the IRR, which operationalizes the contents of the law. Thus, while the law and IRR were congruent, implementation reflected in the annual ARs was not congruent with either.

**TABLE 4.** Annual Trends and Issues Identified in RPRH Implementation^a^

		**Years Identified**				
**Area**	**Issues**	2014	2015	2016	2017	2018
Cross-cutting	No overall plan or a single agency solely in charge of nationwide implementation	◉	◉	◉	◉	◉
	Weak M&E and data management	◉	◉	◉	◉	◉
	Weak link between demand generation and service provision; weak logistics system	◉	◉	◉	◉	◉
	Limited scope and scale of service delivery through public sector	◉	◉	◉		◉
	Capacity-building efforts of NGAs like DOH are limited to public sector	◉	◉	◉		◉
	Uncertainty of RPRH budget; limited absorptive capacity for incremental budgets	◉	◉			
	Low utilization of RPRH benefits; lack in clarity for reimbursements and guidelines	◉	◉	◉	◉	◉
MNCHN	Limited access to services and stagnant/high MMR and IMR due to preventable causes	◉	◉	◉	◉	◉
	Poor newborn, infant, child health and nutrition		◉	◉		◉
FP	High unmet need varying across population groups; LGU difficulty operationalizing FP SDN	◉	◉	◉	◉	
	Variable training standards and requirements for FP licensing or accreditation; few HHR in facilities for competing priorities		◉			
	Legal barriers to providing FP (i.e., TRO)		◉	◉		
	Impractical FP targets and planning, including resolution of bottlenecks				◉	◉
ASRH	Lack of clear legal authority and evidence-based technical guidelines to direct ASRH programs and strategies		◉	◉	◉	◉
	Unavailability of routinely collected age and sex disaggregated data on health service utilization		◉	◉		◉
	Delay in adoption of CSE in K-12; limited IEC on ASRH for parents; ineffective awareness campaigns to raise demand for ASRH services			◉	◉	◉
	High unmet need of adolescents; minors need parental consent to access FP services; lack of youth-friendly treatment centers; stigma					◉
VAWC	Laws with dated or discriminatory content; gaps in local policies to address VAWC or GBV		◉		◉	◉
	Inadequate research and monitoring for GBV- and gender-responsive services		◉			◉
	Lack of comprehensive package of services for survivors (psychosocial, legal, and support)		◉			◉
	Unaddressed cases and slow access to justice		◉	◉		◉
	Lack of service provider capability (barangay VAW desks, WCPU in hospitals)		◉			◉
	Prevention of VAWC is not a priority					◉
STI-HIV/AIDS	Continuing growth of HIV epidemic; rising cases among children (vertical transmission)		◉		◉	
	Limited access to HIV/STI services and info		◉	◉		◉
	Lack of data and research on HIV		◉	◉		
	Lack of laws to protect key populations from discrimination and stigma				◉	

Abbreviations: ASRH, adolescent sexual and reproductive health; CSE, comprehensive sexuality education; DOH, Department of Health; FP, family planning; GBV, gender-based violence; HHR, health human resources; IEC, information, education, and communication; IMR, infant mortality rate; LGU, local government unit; M&E, monitoring and evaluation; MMR, maternal mortality rate; NGA, national government agency; RPRH, Responsible Parenthood and Reproductive Health; SDN, service delivery network; STI, sexually transmitted infection; TRO, temporary restraining order; VAW, violence against women; VAWC, violence against women and children; WCPU, women and child protection unit.

^a^Sources: Annual accomplishment reports 2014–2018.

Persistent challenges and recommendations that were identified highlighted the absence of legal, financial, and M&E mechanisms to support agencies’ initiatives across all KRAs.

#### NIT Challenges in Governance

Despite being aware of these challenges, NIT has not been able to steer NGAs toward higher-level governance functions for a decentralized government. NIT meetings instead focused on reviews and revisions for specific policies (70%), FP logistics and inventory issues (58%), and DOH’s RPRH communication and health promotion programs (51%), which could be handled by program managers within NGAs (Table 5). The meeting agenda over the years also underscored the biomedical approach to RPRH employed by national-level planners.

**TABLE 5.** NIT Agenda and Their Frequency of Discussion^a^

**Agenda: Areas of RPRH Implementation**	**2014** **(n=3)**	**2015** **(n=12)**	**2016** **(n=21)**	**2017** **(n=16)**	**2018** **(n=13)**	**2019** **(n=6)**	**Total** **(n=71)**
Policy reviews and revisions 19 on proscription on abortion and management of complications (DOH AO 2018-03) 5 on requiring an ambulance for hospital licensing (DOH AO 2018-01) 5 on PhilHealth accreditation of stand-alone FP clinics (Circular 2018-05)	2	4	17	10	10	5	48
FP logistics Supply chain management issues (e.g., stock-outs), use of remaining progestin subdermal implants given SC TRO, and inventory counts	—	8	11	7	11	4	41
RPRH communication and health promotion National FP Conference, events, DOH-HPCS presentations on communication plan	—	3	9	10	11	3	36
Monitoring and evaluation FP Form 1, Annual report, data requests	—	8	15	6	4	1	34
Legal restrictions - SC-TRO	—	—	3	13	9	—	25
CSO funding Process of accreditation of grant funding	—	—	4	6	10	5	25
Capacity building for health care providers Training for FP, MNCHN, interpersonal communication and counseling; Accreditation of Training providers	—	3	4	6	5	5	23
Accreditation of health care providers Standardized certification programs and accreditation of CSOs and private providers	—	3	4	6	5	5	23
RPRH service delivery Various discussions on quality and access	1	4	3	6	8	—	22
PhilHealth claims/reimbursements	—	4	5	6	3	3	21
RPRH budget DOH budget cuts, augmentation, convergence budgeting	—	3	7	4	2	1	17
Sorsogon “Pro-Life City” (LGU issue)	—	4	9	2	2	—	17
ASRH technical working group NIT organizational challenge: Functionality and leadership	—	9	—	5	2	—	16
Regional implementation teams Organizational challenge: Functionality, reporting issues	—	1	8	—	4	2	15
Service delivery network PhilHealth facility accreditation and issues on DOH facility standards (e.g., need for ambulance)	—	5	5	1	2	—	13
Quantity of health care providers Deployments for nurses and family health associates	—	1	4	1	6	—	12

Abbreviations: AO, administrative order; ASRH, adolescent sexual and reproductive health; CSO, civil society organization; DOH, Department of Health; FP, family planning; HPCS, Health Promotion and Communication Service; LGU, local government unit; MHCHN, maternal, neonatal, and child health and nutrition; NIT, National Implementation Team; PhilHealth, Philippine Health Insurance Corporation; RPRH, Responsible Parenthood and Reproductive Health; SC, Supreme Court; TRO, temporary restraining order.

^a^Source: NIT meeting transcripts 2014–2018.

Four obstacles may exacerbate NIT’s difficulties performing its multisectoral governance functions.

1. Some of the recurring agenda could have been addressed within 1 or 2 agencies outside NIT, such as CSOs’ requesting funding via agreements with individual agencies. Most glaring are the regular updates on the stock of FP commodities, which could be resolved with a transparent tracking system made available to all NFFP supply chain co-managers.
2. Attempts to foster multisectoral coordination did not have a strategic preplanned meeting agenda; instead, each NIT meeting picked up from the content of the previous meeting. Thus, although 17 meetings broached NGA budgets for RPRH, these did not result in convergence budgeting or a unified financial plan because a clear output was not envisioned or expected.
3. NIT and NGAs did not have the tools to enforce their national-level plans. From 2015 to 2018, 17 meetings were devoted to the “Sorsogon issue,” when a mayor of Sorsogon City restricted the distribution of FP devices in health facilities and limited the FP counseling sessions to natural methods. NIT found that it did not have the necessary investigation mechanisms and protocols to hold the local chief executive accountable. However, new NIT guidelines such as assigning an investigatory body, standards for LGU noncompliance, and appropriate sanctions or responses did not result from these discussions.
4. NIT members also do not have the means to hold each other accountable for meeting their respective targets and to foster more interagency collaboration. Two factors induce compartmentalization: (1) because NIT does not have its own independent staff, each NGA represents itself and finds it difficult to bring up their shortcomings, leading to a lack of corrective action taken even when governance issues are raised, and (2) because each agency sets its implementation targets and reports its accomplishments,^31^ the M&E system prioritizes success indicators, rather than accounting for how different agencies’ programs interface together or how they translate in LGU implementation.

Four obstacles may exacerbate NIT’s difficulties performing its multisectoral governance functions.

Although implementers can formally issue a joint memorandum circular that may integrate the functions of and split accountability between 2 or more NGAs, these were not maximized. Of the 104 reported RPRH policy infrastructures in the annual ARs, only 2 were joint memorandum circulars; both included the DOH as a signatory.

#### Absence of NIT Leadership

The 4 obstacles highlight the absence of clear leadership within NIT. Although the position of chair was assigned to the secretary of health, the pivotal role was initially passed to a former secretary of health who was a private individual at the time. The chair prioritized NGAs’ meeting their program targets and promoted the involvement of CSOs; however, a macro-operational view of RPRH was overlooked in the process. Only later was the position taken on by a DOH official. This set the tone for NIT’s prevailing operating procedure: NIT is expected to resolve programs’ operational issues as they are raised rather than proactively set the multisectoral agenda. Consequently, there is a dearth of attention to and effort for building integrated interventions and the investments required to implement them, which some respondents observed.

The study revealed a dearth of attention to and effort for building integrated interventions and the investments required to implement them.

*The idea of creating the NIT was good. It should be an avenue for the stakeholders to come in together to provide recommendations … But what really happens, it became a venue for partners to air their rants, their complaints, their concerns. So, it doesn’t seem like a recommendatory policy body.* —Respondent 17

Current operations stand in contrast to NGAs’ united effort in the past to lift the status quo ante order and temporary restraining order. Meeting minutes indicate that together, health-sector NGAs, multilaterals, and CSOs advocated for RPRH, provided RH services, and mobilized civil society’s demand for RH until the law and its mandates were declared constitutional. Thus, NIT shows great potential as a venue for stewardship for the full and holistic implementation of the law; however, this outcome has not yet been realized given its focus on specific complaints. The most blatant example of micro-operational thinking is that implementers have still been unable to produce the mandated comprehensive package of RPRH services. Various RPRH services are still demarcated by the units that pilot them, translating to how they are presented as an individual NGA’s accomplishment in the annual ARs. Subsequently, this mindset obscures absent services needed in the bigger picture. However, some RPRH implementers have become aware of this problem and the need for multi-agency collaboration that understands the interplay of different programs.

*It seems like even if this element is connected to reproductive health, more often than not, we do not see the connection. I observe we deal on matters separately so when you say reproductive health, the discussion is confined to them [health agencies]. But for the other elements, we also admit that we sometimes view them without the health lens when actually we should also see, for instance, eliminating violence against women by access to reproductive health.* —Respondent 2

As more implementers shifted to a multisectoral perspective for RH, NIT initially intended to develop a concrete multisectoral implementation plan in 2020. However, their strategy meetings were stalled due to the COVID-19 pandemic.

## DISCUSSION

Based on results presented in the Findings section of this article, we chose the Centers for Disease Control and Prevention policy process (Box) to guide the explanation and interpretation of the governance trends in this section.

BOX **The Centers for Disease Control and Prevention Policy Process**

The process (Figure) includes agenda setting and planning activities before policy implementation; its focus on stakeholder engagement and collaboration is appropriate given the multi-agency nature of RPRH implementation.

**FIGURE:**
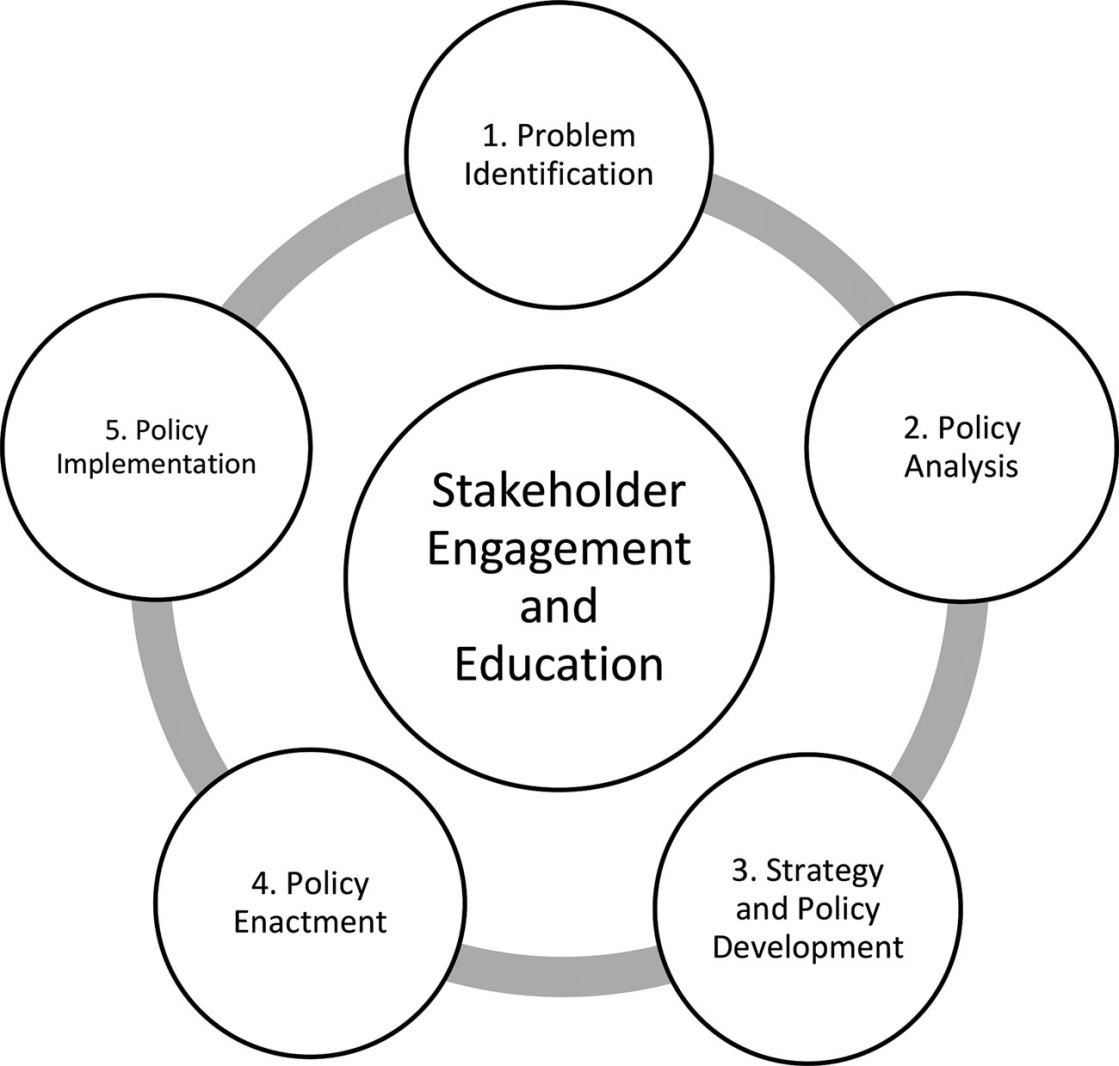
The Centers for Disease Control and Prevention Policy Process^21^

In the problem identification phase, decision makers first agree on the main issue to be addressed, including which populations are most affected, the scale and scope of the issue, and its causes. The framing of the problem narrows the menu of solutions to be considered in the policy analysis phase. The goal of this second phase is to identify, evaluate, and compare different policy solutions according to their (1) effectiveness, defined by how well they respond to the needs of the affected; (2) efficiency, defined as how benefits can be maximized and costs minimized; and (3) feasibility, defined by whether resources are sufficient.

The strategy and policy development phase refers to making the policy actionable, planning for partnerships, and finalizing the form, language, and policy content so the policy is formally and informally accepted. In policy enactment, approval for implementation is obtained from individual and organizational stakeholders.

Finally, the policy implementation phase involves carrying out the main activity or service as well as performing governance functions: establishing the structures, processes, and environment for the sustainability of implementation. These functions comprise educating stakeholders, improving operations and systems, channeling human and financial resources, and constantly monitoring to hold each implementer accountable. At each phase, evaluations determine whether activities answer the policy problem.

The study sought to present and explain trends in national-level implementers’ governance activities to facilitate the implementation of the Philippines’ RPRH Law. It found that implementation focused on the success of specific programs without a clear plan for integrated services to improve the country’s RH outcomes. These programs were heavily skewed toward FP and other biomedical interventions handled by the health sector. Thus, despite the multisectoral nature of RH, implementation efforts and responsibilities were siloed within NGAs and the units that pilot them.

Despite the multisectoral nature of RH, implementation efforts and responsibilities have been siloed within NGAs and the units that pilot them.

However, policy implementers in the Philippines have begun to recognize RPRH is multisectoral. As a country in the first decade of implementing a national RH policy, the Philippines’ challenges to improve RPRH performance are not unique. Governance issues remain major challenges in RH implementation worldwide, particularly among LMICs, and these issues reinforce compartmentalized performance.^32^ Lessons learned from other LMICs’ attempts to coordinate multisectoral action can provide possible governance solutions to improve RH outcomes and promote human development in the Philippines and other neutral policy settings, where there are no clear conflicts of sectors’ objectives.

### Sector-Based Framing

Siloed implementation may be traced to the problem identification phase of RPRH implementation, where implementers painted the issue as the responsibility of discrete NGAs rather than a problem requiring multi-agency solutions. Thus, RPRH programs are inserted wherever they align with existing NGA functions rather than as a part of a prepared multisectoral strategy.

Siloed implementation may be traced to the problem identification phase of RPRH implementation.

In framing the problem, implementers turned to the language of the RPRH Law and IRR; however, these documents do not provide a concrete vision for multisectoral coordination. Although the rules of the RPRH-IRR Drafting Committee identified NIT agencies, local-government coalitions, and CSOs as its members, mandates were more detailed for DOH tasks while tasks of other agencies remained vague. This finding implies that siloed, DOH-focused implementation predated the drafting of the IRR and was not addressed by it. RPRH policy problems are thus framed by individual NGAs and the sectors they represent, which limits policy solutions to a sector’s expertise. Subsequently, agencies unilaterally design, plan, and implement policies to fulfill their mandates without consulting with or studying the effect of other agencies’ RPRH policies.

Non-health sector implementers often are unaware of the extent of the problem,^9^ and a siloed understanding of the problem can hinder coordinated efforts that benefit multiple sectors. In 2013, Ethiopia attempted a multi-agency National Nutrition Program.^33^ Although each sector agreed that undernutrition, food insecurity, and micronutrient deficiencies were pressing issues, the sectors disagreed about their causes, leading to different priorities for advocacy and budget allocation, leading to a lack of multisector plans.

Concurrently, NGAs have vested interests in their own sectors and agencies’ performance. The prevailing tendency to prioritize one’s own sector was also observed in Zambia,^34^ where the government’s maize industry protections may factor into low dietary diversity and the absence of integrated nutrition programs requiring the cooperation of the agricultural and economic sectors.^35^ NIT does not face competing sectoral interests to the same extent. Nonetheless, agencies’ weak coordination is coupled with a reluctance to disclose their implementation shortcomings. As was found in the KIIs, NGA representatives feel they must defend the image of their agency in NIT. Such an environment prevented constructive feedback for perennial agenda items such as the NFFP supply chain. DOH was not incentivized to streamline coordination among its units, and waste could have been avoided by clearly delineating the roles of each agency to cover all necessary activities across the supply chain while having a transparent monitoring and tracking system accessible to all implementers. Thus, limitations of the RPRH Law and reliance on health sector leadership have failed to provide a case for investing in what is still perceived as a health issue, over the inclination toward their own sectors’ concerns.

However, the creation of a single compelling narrative can engage different sectors and maintain continuity despite leadership changes. Peru’s^34^ national nutrition programs successfully achieved their targets because of the narrative to eradicate under-5 malnutrition by 5% in 5 years. The 2006 advocacy campaign was so popular among Peruvians that officials across agencies, parties, and presidential terms were inclined to focus on nutrition.

Multisectoral framing in policy planning more accurately responds to the problems faced by health systems.^9^ Other sectors’ involvement in public health policies become more apparent when considering the social determinants of health,^36^ which highlights that the nonmedical context of people’s daily lives influences their health outcomes. By making clear how each agency understands a problem and can contribute to a solution, a holistic approach to policy making can be undertaken.

By making clear how each agency understands a problem and can contribute to a solution, a holistic approach to policy making can be undertaken.

### Health-Centric Policy Options

Of the 5 health-centric KRAs, FP deliberately received the most attention from implementers to compensate for the political challenges FP programs faced. Preoccupation with these legal battles^37^ may have hampered attempts to comprehensively build a multisectoral strategy beyond FP. The emphasis on biomedical programs and FP may be analyzed according to the issues faced by public administrators during the policy analysis and strategy development phases.^21^

FP is a national priority at multiple levels of governance (e.g., Office of the President, NIT, NGAs), and the continual discussion of a particular agendum primes strong opinions and decisions in favor of engaging with the issue.^38^ Although fertility management can indeed aid in improving health outcomes, a predisposition to FP interventions precludes exploring how other RPRH elements can supplement them and address other RH policy problems.

The prominence of FP interventions exemplifies the biomedical paradigm, in which “clinical interventions take on aggressive forms and emphasize short-term results,”^39^ based on the premise that the body can be treated independently of one’s context.^40^ Consequently, nonmedical and preventive interventions, which address the impacts of the individual’s socioeconomic, political, and environmental context, are viewed as less legitimate. Such a paradigm individualizes the issues that may be caused by systemic or structural inequalities, and poor social, economic, and health outcomes are attributed to individual failure.

Although many countries’ development paradigms once relied heavily on managing fertility through biomedical interventions, a focus on the quality of life afforded each citizen emerged following the 1994 Cairo Declaration on Population and Development.^41^ Since then, countries that famously prescribed population control, like China,^42^ have begun to focus on improving social services while easing restrictions on family size.

Thailand’s decentralized government^43^ also faced difficulties providing integrated and coordinated RH services across sectors.^44^^,^^45^ This situation changed in 2017 when the government recognized that underlying factors such as better education, better jobs, and higher income, especially among women, led to greater gains in reducing the total fertility rate than their NFFP alone could have achieved.^46^ Thai RH policy then shifted to providing a better-quality life to those born by basing their success and impact indicators on human rights and the social determinants of RH, similar to the United Nations Population Fund’s demographic dividend index,^47^ which integrated various human capital inputs for health, employment, youth rights, and education to track African states’ progress toward sustainable development.

Should the Philippines employ a similar index, various RPRH can be connected congruent with the vision of the RPRH Law and IRR. The index can provide objective annual benchmarks to hold NIT members accountable, while also engaging with non-health sectors.

Through empowering non-health sectors in NIT, more holistic policy options for RH can be devised for the needs of the Philippine RH context. Meaningful participation of different sectors can provide expert information on solutions that may not have been apparent to a sector alone.^21^ Some examples include reforming the private insurance market in the state of California in the United States to automatically include comprehensive maternity benefits for any health care plans.^48^ US federal policy also integrates health care, criminal justice, and social work policies to address sexual and partner violence.^49^ As more studies highlight the vulnerability of disadvantaged groups to poor RH outcomes,^50^^–^^52^ understanding the dynamics of one’s socioeconomic, political, and community context is more relevant than ever.

Through empowering non-health sectors in NIT, more holistic policy options for RH can be devised for the needs of the Philippine RH context.

### Weak Leadership for Implementation

In RPRH implementation, NIT serves as the coordinating body for RPRH activities. However, persistent governance challenges in planning, financing, monitoring, and accountability, may be attributed to the structure and activities of NIT during the policy enactment and policy implementation phases.

NIT’s leadership role is fundamental to engaging with different sectors. Effective leadership can coordinate stakeholders and overcome opposition and resource deficits. For instance, health interventions to control noncommunicable diseases are often met with pushback from the food, tobacco, or alcohol industries.^9^ However, in Turkey, Brazil, and Norway,^9^ stewardship catalyzed policy creation, united different sectors to pressure industries, and eventually shifted the policy environment to produce better health outcomes.

NIT’s leadership role is fundamental to engaging with different sectors, and effective leadership can coordinate stakeholders and overcome opposition and resource deficits.

Effective leadership is tied to 3 accomplishments^21^:

1. Outcomes and goals must be made clear to all stakeholders. Since LGUs are ultimately responsible for RPRH service delivery, local chief executives need to be educated and empowered.
2. Policy makers must identify the necessary resources for implementation. These include not only the financial and human resources needed to execute the programs but also the policy infrastructure to support changes made to systems that ensure the sustainability of implementation.
3. Implementers must designate who is involved and what each actor’s responsibilities are in a concrete plan, which includes having a clear leader, definite roles for each implementer, and plans with opportunities for collaboration in mind.^53^

To address such challenges, NIT can reform its structure. The present NIT structure is flat, with no agencies having authority over others. One alternative model would appoint a dedicated full-time NIT executive team to create guidelines and incentives, while serving as an objective arbiter holding members to their benchmarks. Historically, POPCOM operated under such an arrangement, before its functions were diminished during its subsumption into DOH and later back to the National Economic Development Authority.^54^^–^^56^

Another model involves restructuring NIT into management teams^57^ or committees. Currently, NIT has technical working groups, each composed of different agency representatives; however, meeting transcripts reveal an absence of their contribution to NIT operations. Future research may explore optimal arrangements for NIT technical working groups, coordinated by the chair, as the main driver for NIT’s governance activities.

Such reforms within NIT will facilitate multi-agency policy integration, changing administrative practices and institutional culture and assimilating RH into different policies.^58^ Policy integration weaves RH into all levels of an agency’s agenda (vertical integration) and across sectors (horizontal integration). Rather than aiming to create 1 policy executed by each actor, the goal is to study the relationships of each agency’s policies and how they can address the issue together.

Reforms within NIT will facilitate multi-agency policy integration, changing administrative practices and institutional culture and assimilating RH into different policies.

As evidenced in NIT, however, many LMICs face the barrier of the traditional hierarchical structure of state organizations,^9^ which tends to value vertical authority over horizontal partnerships. Through horizontal planning and communication, NGAs can veer away from program-specific operational challenges toward creating formal partnerships and identifying areas where multisectoral dimensions can be added to pre-existing policies, programs, and indicators.^59^ Planning also promotes accountability,^60^ which fosters ownership and commitment to carrying out NGAs’ roles.

Together, these trends may have contributed to slow improvements in RH outcomes despite the law’s passage. In 2015, the country failed to meet its Millennium Development Goals for reducing maternal mortality, lowering HIV/AIDS incidence, and eradicating poverty.^61^ Currently, the country stands to once again fail to meet its Sustainable Development Goals as the maternal mortality ratio remained at 121 maternal deaths for every 100,000 live births in 2017,^62^ Philippine HIV infections are one of the fastest growing in Asia,^63^ and the poorest Filipino women are 5 times more likely than the richest income quintile to have begun childbearing in their teenage years.^64^

### Limitations

The results of this research should be considered in light of 3 limitations. Because the study focused on national-level governance, the scope of KIIs was limited to national-level implementers. The impact of NGAs on subnational government units’ RPRH activities could not be assessed, and due to the sudden outbreak of COVID-19 disease, interviews with every single implementing agency were not pursued. Given the focus on trends in governance, KIIs also did not cover the specifics of each RPRH program and policy and governance of discrete programs such as supply chains in depth, as those warrant separate nuanced studies. Nonetheless, data collection for the KIIs had reached a point of saturation and common themes could be identified from the interviewees’ responses. Moreover, other data collection techniques such as official document reviews and literature searches were employed when necessary to provide more context for NGAs’ tasks, policies, and programs. Despite religious groups’ visible presence in the history of the law, their noninvolvement in national governance for its implementation precluded them from the scope of this study. Additionally, their role in the law’s implementation at the national level was not mentioned in our interviews or reviews of documents such as the annual ARs. Although some religious groups may aid in the implementation of the law at the community level, their involvement was beyond the national-level scope of this study.

Except for NIT meeting transcripts, document reviews included only published documents, reports, and policies. Of the official documents, quantitative success indicators reported by NGAs were not analyzed. Rather, as the first study to assess national-scale RPRH performance, the study was focused on trends in performance to identify major areas for administrative reform.

Finally, given the nature of this study as part of the first review of RPRH implementation, literature on multi-agency RPRH efforts in the Philippines was scarce. As such, the study looked for parallels of multisectoral action for health in other LMICs. The study also revealed gaps in prior literature on comprehensive multisectoral RH policies, particularly in LMICs, as most pertained to nutrition programs.

## CONCLUSION

As more developing countries formally recognize the need for multidimensional interventions to improve the lives of their citizens, multisectoral governance becomes ever more crucial to create strategies and enforce commitments across government agencies. While comprehensive studies on multisectoral policies in LMICs are scarce, those on RH are even more so. This study sought to extend the literature as part of the first evaluation of the Philippines’ RPRH Law.

The Philippines’ implementation of the RPRH Law presents a case of a country in the first decade of implementing a multisectoral initiative for sustainable human development. Agencies’ viewing only the interests and interventions under the purview of their respective sectors has limited interagency collaboration. Although RH is multidimensional, historical focus on the law’s fertility management aspect has skewed policy options toward biomedical interventions. Despite having an interagency coordinating body, NIT has not reached its full potential, including creating a multi-agency strategy for RPRH implementation. Faced with a lack of formal mechanisms to facilitate multi-agency coordination, NGAs’ implementation of the law is fragmented, biomedical, and programmatic and has not led to remarkable changes in the country’s RH status.

In light of these challenges, policy makers have begun to acknowledge the importance of an integrated and coordinated response to modern RH issues.

Although multisectoral governance challenges are complex, experiences from other LMICs can illuminate possible actions to address them. Creating a single compelling narrative to frame the policy issue can emphasize the multidimensional nature of the problem and identify structural causes, which may not straightforwardly relate to health. Including different sectors’ expertise, through horizontal integration and communication, broadens policy solutions and provides more benefits to populations. Developing a single multisectoral index to benchmark each implementer’s annual progress can promote accountability. Finally, appointing a dedicated RPRH lead while assigning sector representatives to committees may be a more effective model of governance that promotes planning and accountability.

## Supplementary Material

21-00184-Siy-Van-Supplement.pdf

## Notes

**Peer Reviewed**

**First published online:** September 8, 2021.
