# Supplementary material for: Trends in National-Level Governance and Implementation of the Philippines’ Responsible Parenthood and Reproductive Health Law from 2014 to 2020
Source: Glob Health Sci Pract. 2021 Sep 30;9(3):548–64. doi: 10.9745/GHSP-D-21-00184 (PMC8514023; doi:10.9745/GHSP-D-21-00184)
Supplement: 21-00184-Siy-Van-Supplement.pdf [file 21-00184-Siy-Van-Supplement.pdf]

Supplement to: Siy Van VT, Uy J, Bagas J, Ulep VGT. Trends in national-level governance and implementation of the Philippines' Responsible Parenthood and Reproductive Health Law from 2014 to 2020. *Glob Health Sci Pract*. 2021;9(3). https://doi.org/10.9745/GHSP-D-21-00184

Supplement

Supplement A. Organization of the Philippine Government and its RH Law

The Philippines has a unitary democratic government, with national-level agenda setting and legislation. Government and social services are decentralized as instituted by the Local Government Code of 1991:[1] political subdivisions of the government are given autonomy to prioritize policies, allocate resources, and hire manpower to meet the most pressing needs of their subnational jurisdictions. At the central government level, 21 executive departments create national-scale policies, programs, and plans. These are conveyed to their regional counterparts, who convey program guidelines to local government units (LGUs) and collect updates on their progress. LGUs refer to government offices from the provincial level to the city or municipality and then to the *barangay*, the smallest political administrative division in the country. Thus, centrally designed policies are ultimately implemented through LGUs, which have discretionary power over the prioritization of programs dispatched by the central government.

Within this government structure, the Philippines passed the Responsible Parenthood and Reproductive Health (RPRH) Law[2] in 2012, declaring universal access to RH services as integral to the rights to life, health, and sustainable human development. Its ratification overcame years of opposition from conservative groups, including some representatives of the Catholic Church who viewed contraception that prevented implantation after ovum fertilization as an abortifacient,[3,4] and presented a shift in the way the Philippine state viewed the role of women, the family, and RH in poverty alleviation and the socioeconomic development of the nation. Through RH, families could care for and invest in all their members, contributing to a healthier, happier, more productive Philippine population.

The RPRH Law defined RH care as having 12 elements ranging from family planning (FP) to sexuality and RH education and the elimination of gender-based violence. These acknowledged the multi-actor nature of RPRH, including national government agencies (NGAs), private sector, civil society organizations (CSOs), multilaterals, and LGUs (Table S.1). Consequently, good governance to facilitate multisectoral coordination became a

---

[1]Republic of the Philippines. An Act Providing for a Local Government Code of 1991.; 1991. Accessed February 21, 2021. https://www.officialgazette.gov.ph/downloads/1991/10oct/19911010-RA-7160-CCA.pdf
[2]Republic of the Philippines. An Act Providing for a National Policy on Responsible Parenthood and Reproductive Health.; 2012. Accessed October 17, 2019. https://www.lawphil.net/statutes/repacts/ra2012/ra_10354_2012.html
[3]Dañguilan MJ. The RH Bill Story: Contentions and Compromises. Ateneo de Manila University Press; 2018. Accessed July 31, 2020. http://www.ateneo.edu/ateneopress/product/rh-bill-story-contentions-and-compromises
[4]Genilo EM. The Catholic Church and the Reproductive Health Bill Debate: The Philippine Experience. The Heythrop Journal. 2014;55:1044-1055. Accessed May 25, 2021. https://jliflc.com/wp-content/uploads/2014/10/heyj12203.pdf

Supplement to: Siy Van VT, Uy J, Bagas J, Ulep VGT. Trends in national-level governance and implementation of the Philippines' Responsible Parenthood and Reproductive Health Law from 2014 to 2020. *Glob Health Sci Pract*. 2021;9(3). https://doi.org/10.9745/GHSP-D-21-00184

determinant for the success of implementing agencies that guide LGU implementation of RPRH interventions in communities.

TABLE S.1. Government Agencies Involved With the Implementation of the Responsible Parenthood and Reproductive Health (RPRH) Law

Agency	Main Responsibility
Department of Health (DOH)	Lead agency for nationwide implementation
Commission on Population and Development (POPCOM)	Co-management of National Family Planning Program with the DOH
Philippine Health Insurance Corporation	Insurance coverage for RPRH services to improve affordability
Food and Drug Administration	Regulation of family planning products and devices
Philippine Commission on Women	Promote gender equality and women's empowerment and address gender-based violence; Co-Secretariat of the National Implementation Team with the DOH and POPCOM
Department of Social Welfare and Development	Integrate RPRH into antipoverty and social welfare programs
National Anti-Poverty Commission	Coordinate with government agencies to identify and target the poor for access to RPRH health care and information services
Department of Interior and Local Government	Communicate and facilitate local government unit compliance with RPRH implementation plans and guidelines
Local government units	Direct service provider of RPRH health and information services
Department of Education	Integrate RPRH into basic education curricula
Commission on Higher Education, Technical Education and Skills Development Authority, Professional Regulation Commission	Include RPRH in degree and nondegree programs and continuing professional development

Source: Author synthesis from Implementing Rules and Regulations of the RPRH Law, 2017 revision.[5]

[5]IRR Drafting Committee for Republic Act No. 10354, Commission on Population and Development. Revised Implementing Rules and Regulations (IRR) of the Responsible Parenthood and Reproductive Act of 2012 (RA No. 10354). Philippine Department of Health; 2017:91. Accessed October 17, 2019. https://www.doh.gov.ph/sites/default/files/basic-page/Final%20Revised%20Implementing%20Rules%20and%20Regulations%20%28IRR%29%20of%20RPRH%20Law_0.pdf

Supplement to: Siy Van VT, Uy J, Bagas J, Ulep VGT. Trends in national-level governance and implementation of the Philippines' Responsible Parenthood and Reproductive Health Law from 2014 to 2020. *Glob Health Sci Pract*. 2021;9(3). https://doi.org/10.9745/GHSP-D-21-00184

Supplement B. Respondent Characteristics

Respondents included the chair of NIT as well as the executive director and deputy executive director of a government agency. Division chiefs of NGAs (3, 15%), an assistant division chief, and a team leader were also interviewed in addition to program managers or supervising program specialists (8, 40%). Given the decentralized nature of the Philippine government, representatives in (10%) regional positions were also interviewed. One CSO represents the private sector in NIT meetings, and its executive director and founder of 25 years was interviewed. A representative from a multilateral agency involved in RPRH was also interviewed. Of these respondents, 13 (65%) respondents were women and 7 (35%) were men.

Supplement to: Siy Van VT, Uy J, Bagas J, Ulep VGT. Trends in national-level governance and implementation of the Philippines' Responsible Parenthood and Reproductive Health Law from 2014 to 2020. *Glob Health Sci Pract*. 2021;9(3). https://doi.org/10.9745/GHSP-D-21-00184

Supplement C. Responsible Parenthood and Reproductive Health Elements with Major Programs Reported in Annual Accomplishment Reports 2014-2018

TABLE S.2. Responsible Parenthood and Reproductive Health (RPRH) Elements with Major Programs Reported in Annual Accomplishment Reports (ARs) 2014–2018

RPRH Element	Agency	Programs	Total
1. Family planning information and services	DOH	1	4
	POPCOM	2	
	DOLE	1	
	DSWD	1	
2. Maternal, infant, and child health and nutrition, including breastfeeding	DOH	3	8
	DSWD	2	
	DepEd	1	
	DOLE	1	
	CSO	1	
3. Proscription and management of abortion and its complications	None reported in the ARs		
4. Adolescent youth and reproductive health guidance and counseling at the point of care	CSOs, POPCOM, UNFPA, USAID, Private	1	3
	DepEd	1	
	DOH	1	
5. Prevention, treatment, and management of reproductive tract infections (RTIs), HIV and AIDS and other STIs	DOH	3	8
	DSWD	1	
	CSO	4	
6. Elimination of violence against women and children and other forms of sexual and gender-based violence	DSWD	2	15
	POPCOM	1	
	DOH	1	
	CSO	3	
	PCW	5	
	PNP	1	
	DILG, LGU	2	
7. Education and counseling on sexuality and reproductive health	DOH Region 7	1	7
	NAPC	1	
	POPCOM	5	
8. Treatment of breast and reproductive tract cancers and other gynecological conditions	DOH	2	2
9. Male responsibility and involvement in RH	POPCOM	1	2
	DSWD	1	

Supplement to: Siy Van VT, Uy J, Bagas J, Ulep VGT. Trends in national-level governance and implementation of the Philippines' Responsible Parenthood and Reproductive Health Law from 2014 to 2020. *Glob Health Sci Pract*. 2021;9(3). https://doi.org/10.9745/GHSP-D-21-00184

10. Prevention, treatment and management of infertility and sexual dysfunction	None reported in the ARs		
11. Reproductive health education for adolescents in formal and non-formal education settings	DSWD	3	13
	National Youth Council	1	
	DepEd	6	
	POPCOM	2	
	DOH	1	
12. Mental Health Aspect of Reproductive Care	DOH	1	1

Abbreviations: CSO, civil society organization; DepEd, Department of Education; DILG, Department of Interior and Local Government; DOH, Department of Health; DOLE, Department of Labor and Employment; DSDW, Department of Social Welfare and Development; LGU, local government unit; NAPC, National Anti-Poverty Commission; PCW, Philippine Commission on Women; PNP, Philippine National Police; POPCOM, Commission on Population and Development; UNFPA, United Nations Population Fund; USAID, United States Agency for International Development.

Sources: Annual ARs, 2014–2018.
